# Recognizing Hemiparetic Ankle Deficits Using Wearable Pressure Sensors

**DOI:** 10.1109/JTEHM.2019.2942922

**Published:** 2019-10-08

**Authors:** Ahmed Ramadan, Anindo Roy, Elisabeth Smela

**Affiliations:** 1 Department of Physical Therapy and Rehabilitation ScienceUniversity of MarylandBaltimoreMD21201USA; 2 Department of NeurologyUniversity of MarylandBaltimoreMD21201USA; 3 Department of Mechanical EngineeringUniversity of MarylandCollege ParkMD20742USA

**Keywords:** Piezoresistive, wearable assistive robot, lower extremity therapy, microcontroller

## Abstract

Objective: To provide proof-of-concept for a novel method to recognize impaired push-off and foot-drop deficits in hemiparetic gait using analog pressure sensors. These data may enhance feedback from a modular ankle exoskeleton (such as Anklebot) for stroke rehabilitation, which now employs on/off foot switches under the foot. Methods: A pressure sensor was positioned on the posterior side of the calcaneus. Experiments were conducted on two healthy subjects with normal walking and with hip circumduction and foot drop, the latter to mimic hemiparetic gait post-stroke. Results: Unlike the foot switches, the pressure sensor yielded data during swing. The initial swing and terminal stance readings followed local foot-shoe dynamics and were thus able to detect foot drop swing deficits while also providing push-off information during stance. Discussion: The analog pressure sensors provided more information than foot switches, even during stance. This system may provide clinicians with a tool to monitor foot drop and push-off.

## Graphical abstract

We present a novel method to recognize impaired push-off and foot-drop deficits in hemiparetic gait using analog pressure sensors. The sensors provided more information than foot switches, even during stance. This system may provide clinicians with a tool to monitor foot drop and push-off.

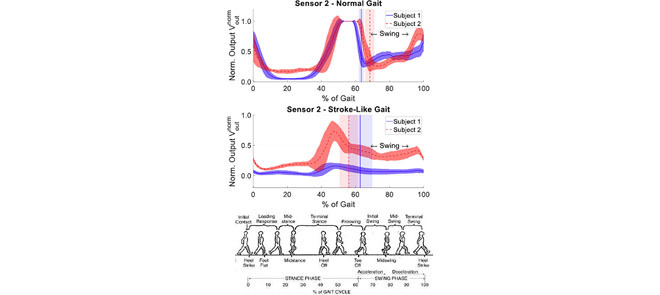

## I. Introduction

Post-Stroke hemiparesis is characterized by impaired motor control on one side of the body. Hemiparetic gait is manifested as impaired ankle motor control, contributing to gait and balance deficits. Ankle deficits occur during either the stance phase (e.g. impaired push-off) or the swing phase (e.g. foot drop). Both limit speed, safety, and symmetry of walking while exacting higher metabolic cost. As a result, stroke survivors face mobility challenges [1].

Lower extremity robots have recently been developed for hemiparetic gait rehabilitation. One such device, a two-degree-of-freedom actuated ankle robot (Anklebot) [2], can provide assistance as needed. To precisely time robotic assistance to gait events corresponding to ankle deficits occurrence, an insole instrumented with four on/off switches at the toe, heel, medial, and lateral portions of the foot is used [3]. Each switch generates a unique non-zero voltage when closed. The resultant encoded voltage has a discrete staircase pattern in which each voltage value corresponds to the portion of the foot that is in contact with the ground.

While discrete kinematic indicators from the foot switches have proven effective in robotic gait therapy [4], one drawback is the absence of any information during a constant-voltage period. For example, while the foot switches accurately and robustly detect toe-off, the voltage is zero throughout swing, thereby not affording any scope to detect deficit-related ankle behaviors such as foot drop. Likewise, the foot switches can detect heel-off as an indicator of late stance but cannot provide any information on ankle behaviors related to propulsion deficits due to the staircase voltage pattern.

For future gait rehabilitation, lower extremity robots should be portable and stand-alone, providing clinicians a tool to remotely monitor (1) foot drop without physical exam or video recordings and (2) push-off without laboratory anchored instrumentation. Wearable pressure sensors were used to measure local pressure signals under the foot as part of instrumented insoles [5], [6]. They were also used to measure dorsal pressure between the foot and the shoe upper for footwear design [7], [8]. Therefore, we hypothesize that it would be feasible to locate pressure sensors around the top and sides of the foot to monitor push-off and foot drop. If true, pressure sensors can be an impactful augmentation to the existing lower extremity robots.

## II. Methods

### A. Human Subjects

Two healthy subjects (males) participated in the experiments. They had no history of neurological motor control impairment or gait-related pain lasting more than a week. Subjects gave informed consent, whose procedures were approved by the University of Maryland Institutional Review Board (IRB), as part of a pre-existing IRB-approved protocol (IRB# 1204238-1; deemed Minimal Risk) that followed the Declaration of Helsinki.

### B. Sensor Setup

**FIGURE 1.**
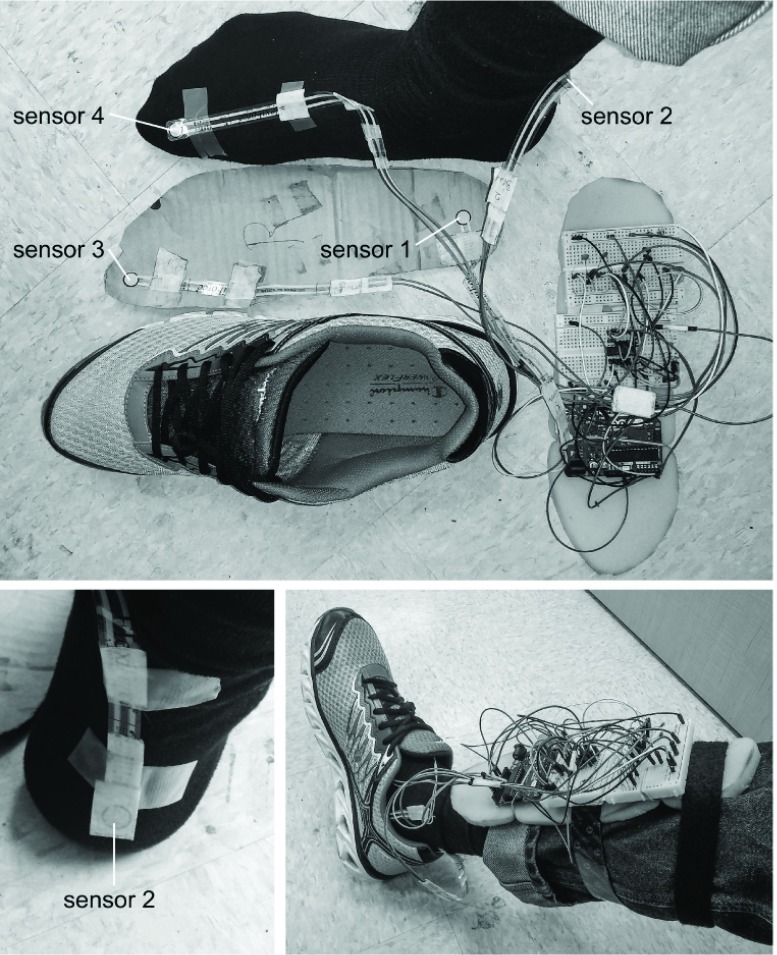
The experimental setup and pressure sensor locations. Top: Four pressure sensors located under the heel, on the posterior side of the calcaneus, under the big toe, and atop the big toe. Bottom Left: Close-up of sensor 2. Bottom Right: Integrated experimental setup.

Four piezoresistive pressure sensors (A201, FlexiForce, Tekscan, Boston, MA) were attached to either the foot or inside the subject’s shoe (Fig. 1). Sensors “1” and “3” were attached to an insole corresponding to positions under the heel and under the big toe to capture ankle behaviors during stance. Sensor “2” was attached to the foot on the posterior side of the calcaneus, and sensor “4” was placed atop the joint of the big toe to capture behaviors during swing.

A motion capture system measured the kinematics of the instrumented foot in subject 2 (see Supplementary Material). The objective was to validate how the subject performed stroke-like walking, and hence the deficits of foot drop and diminished push-off. (A video recording of the stroke-like walking is in the Supplementary Material.)

### C. Data Collection and Analyses

**FIGURE 2.**
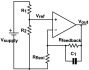
The non-inverting amplifier circuit. The supply voltage $V_{\mathrm {supply}} = 5$ V. The baseline offset $V_{\mathrm {ref}} = 0.25$ V is determined by the values of $R_{1} = 1952\,\,\Omega $ and $R_{2} = 101\,\,\Omega $.$~\,R_{\mathrm {flexi}}$ is the pressure sensor resistance. $R_{\mathrm {feedback}}$ is the resistor that determines the amplifier gain. The capacitor was $C_{1} = 13$ nF.

Non-inverting amplifier circuits converted changes in the resistance of the pressure sensors to voltages proportional to the pressure (Fig. 2). The amplifier model is:

\begin{equation*} V_{\mathrm {out}}=V_{\mathrm {ref}}\left ({1+\frac {R_{\mathrm {feedback}}}{R_{\mathrm {flexi}}} }\right),\tag{1}\end{equation*}

where $V_{\mathrm {out}}$ is the analog output voltage, $V_{\mathrm {ref}}$ is a baseline offset voltage, $R_{\mathrm {feedback}}$ is an external resistance that determines the amplifier gain, and $R_{\mathrm {flexi}}$ is the pressure sensor resistance. When there is no pressure on the sensor, $R_{\mathrm {flexi}} = \infty $ (open circuit) and the output voltage is $V_{\mathrm {ref}}$. With increasing pressure, $R_{\mathrm {flexi}}$ decreases, so $V_{\mathrm {out}}$ increases until saturation at $V_{\mathrm {supply}} = 5$ V. $V_{\mathrm {ref}}$ was set using fixed resistors to 0.25 V. The amplifier gain of each sensor was set by adjusting $R_{\mathrm {feedback}}$(Table I).

**TABLE 1:** $R_{\mathrm{feedback}}$ for Each Walking Condition and Sensor. $R_{\mathrm{feedback}}$ Determines the Amplification Gain (Fig. 2 and Equation 2)

Subject	Walking Condition	$R_{\mathrm {feedback}}\mathrm {(\Omega)}$			
		Sensor 1	Sensor 2	Sensor 3	Sensor 4
S1	Normal	164.1 k	2 M	490 k	6 M
S1	Stroke-Like	490.0 k	4 M	3 M	4 M
S2	Normal	164.1 k	2 M	490 k	NA
S2	Stroke-Like	164.1 k	2 M	490 k	NA

An Arduino Uno (Arduino, Turin, Italy) logged the data ($V_{\mathrm {out}}$), sampled at 200 Hz. A laptop running a custom MATLAB code serially communicated with the Uno via a USB cable. Data were plotted in real time, allowing visual inspection to assure signal fidelity during the trial.

Each subject started each trial with the instrumented foot not touching the ground, subsequently initiating gait with a heel strike on a walkway, at an uncontrolled, self-selected comfortable speed. Data were collected for two conditions: “normal” and “stroke-like”. The latter mimicked hemiparetic gait and consisted of intentional hip circumduction and foot drop during swing followed by foot slap at initial contact. Trials resulting in incomplete data (e.g., due to poor wire connections) were not included in data analysis. Post-hoc analysis with a custom MATLAB code identified and extracted each gait cycle.

Measurement voltages were normalized to a unitless value between 0 and 1.

\begin{equation*} V_{\mathrm {out}}^{\mathrm {norm}}=\frac {V_{\mathrm {out}}-V_{\mathrm {ref}}}{V_{\mathrm {supply}}-V_{\mathrm {ref}}}=\frac {V_{\mathrm {ref}}\left ({\frac {R_{\mathrm {feedback}}}{R_{\mathrm {flexi}}} }\right)}{V_{\mathrm {supply}}-V_{\mathrm {ref}}}\tag{2}\end{equation*}

The denominator was a constant 4.75 V, and since $V_{\mathrm {ref}}$ was held constant, $V_{\mathrm {out}}^{\mathrm {norm}}$ was proportional to $R_{\mathrm {feedback}}$.

The number of data points varied between gait cycles due to variations in step duration. The MATLAB spline function interpolated the data to afford comparison between data sets at the same percentages of the gait cycle. The mean voltage and standard deviation could then be calculated for each walking condition.

## III. Results and Discussion

Sensor 2 yielded repeatable patterns during the swing phase. Measurements from sensor 4 (for subject 1 only) were non-zero only during initial swing, with larger variability. Therefore, sensor 4 data are not presented in this brief. Sensors 1 and 3 detected the start and end of the stance phase. Examples of the measurements from all sensors are in the Supplementary Material.

**FIGURE 3.**
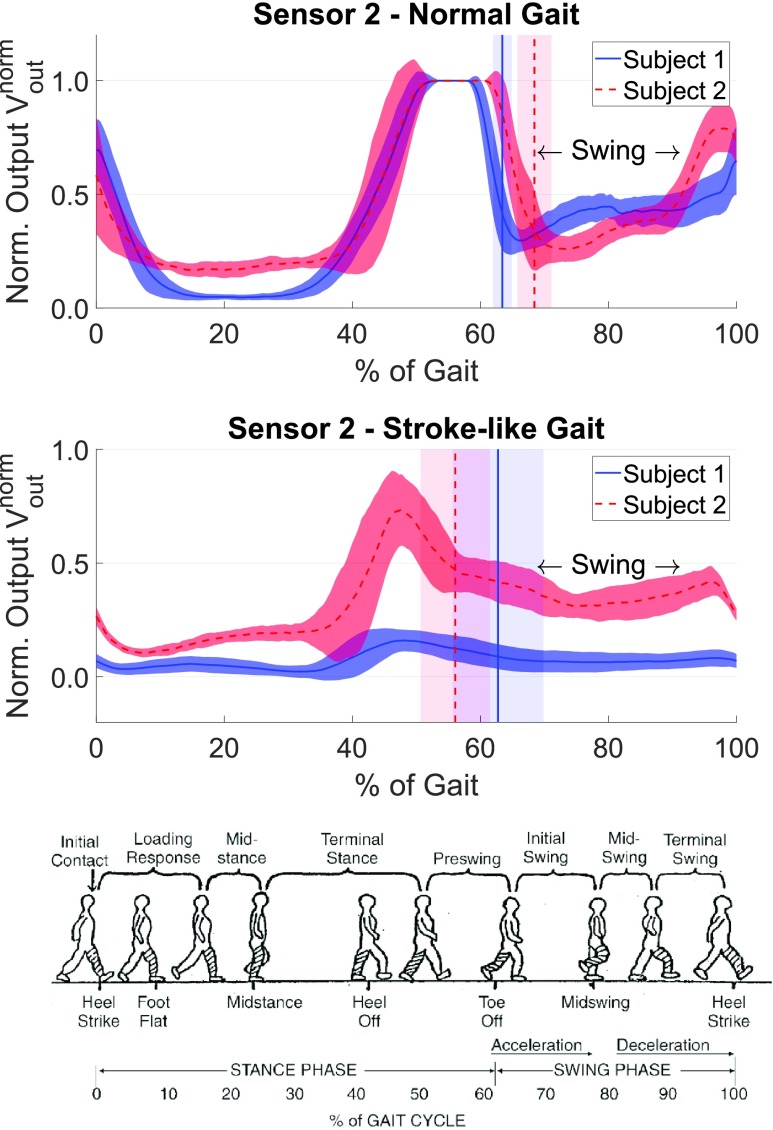
Sensor 2 measurements (mean ±SD) during normal (top) and stroke-like (middle) gait conditions. The vertical lines indicate toe-off or swing start (mean ±SD). Walking conditions with the gait phases (bottom) courtesy of Carson Schneck [9].

Sensor 2 measurements are summarized in Fig. 3 for both subjects. In stroke-like walking, $V_{\mathrm {out}}^{\mathrm {norm}}$ was smaller than in normal walking, especially in subject 1. Also, the measurement variability was larger in stroke-like gait.

During swing (~63–100% cycle), sensor 2 voltages were non-zero. Qualitatively, immediately after toe-off (vertical line) in normal walking the voltage dipped and then gradually increased again. On the other hand, in stroke-like gait the voltage slowly decayed to a plateau, with a small increase towards the end of the cycle. This suggests that the local pressure between the foot and the shoe at sensor 2 experienced more dynamics (acceleration/deceleration) in normal gait compared to stroke-like gait. The pressure dip during initial swing in normal gait may be attributed to the foot’s forward acceleration, resulting in lower local pressure at the heel. In contrast, this pressure decrease was negligible or missing in stroke-like gait. Correspondingly, in normal gait during terminal swing (~90–100% cycle) the foot decelerates before touching the ground, leading to an increase in pressure at the heel. Again, this phenomenon was absent or less dominant in stroke-like gait.

During terminal stance (~25–50% cycle [9]), normal gait showed a steep rise in voltage, reflecting a greater pressure on sensor 2, consistent with push-off. In contrast, stroke-like gait was characterized by a less pronounced voltage increase, consistent with impaired push-off.

## IV. Conclusions and Future Directions

Analog readings from a proximally placed pressure sensor provided enhanced information during the stance phase and new information during the swing phase, compared to the current discrete foot switches. The measurements are consistent with gait biomechanics and hemiparetic deficits. For example, stroke-like gait does not result in appreciable local dynamics between the heel and the shoe. This phenomenon manifests as a plateau-like pressure signal during swing and as a diminished pressure rise during late stance. The use of an analog sensor placed on the heel accurately captured these manifestations.

This novel method is in an early translational development phase, tested with two subjects performing stroke-like walking. Given the small sample size, our findings and their generalizability should be viewed with caution since methodological tweaks may be needed based on clinical data from a larger sample of able-bodied and stroke subjects. Future research will also integrate the dynamic pressure information into the robot’s impedance controller. Overall, this research is expected to: (1) enable clinicians to diagnose foot drop and monitor its recovery without a physical exam; and (2) provide intra-phase robotic assistance beyond that informed by discrete event sensors.
